# Differences between generalized Q-sampling imaging and diffusion tensor imaging in visualization of crossing neural fibers in the brain

**DOI:** 10.1007/s00276-019-02264-1

**Published:** 2019-05-29

**Authors:** Zhuoru Jin, Yue Bao, Yong Wang, Zhipeng Li, Xiaomeng Zheng, Shengrong Long, Yibao Wang

**Affiliations:** 1 Department of Neurosurgery, The First Affiliated Hospital of China Medical University, No. 155, North Nanjing Street, Heping District, Shenyang, 110001 Liaoning P. R. China; 2 Department of Neurosurgery, University of Birmingham, Edgbaston Street, Birmingham, UK

**Keywords:** Diffusion tensor imaging, White matter, Optic nerve, Pyramidal tract

## Abstract

**Purpose:**

The aim of this study was to discuss the advantages of GQI reconstruction in the imaging of nerve fibers at crossing regions. Compared with DTI, the paper also discussed the advantages of GQI in imaging principles.

**Methods:**

3-T MRI data from five normal participants were reconstructed using GQI and DTI. After adjusting the parameters, we compared the differences in reconstructed nerve fibers at the crossing regions between the two methods. To complete this study, we chose four obvious examples (the optic nerve, the Superior cerebellar peduncles, the intersection of the pyramidal tract, the corpus callosum and the arcuate fibers and the intersection of the supplementary motor area (SMA) and the anterior part of arcuate fasciculus) to illustrate.

**Results:**

By reconstructing nerve fibers in three regions, we can find that crossing-area images of nerve fibers significantly differed between DTI and GQI reconstruction. Although crossing fibers could be clearly and completely visualized after GQI reconstruction, they showed artifacts, incompleteness, deletions, and fractures after DTI reconstruction. After GQI reconstruction, we can find that there were two or more nerve fibers in each voxel. However, only one nerve fiber was present in each voxel after DTI reconstruction.

**Conclusion:**

The imaging of crossing fibers is more complete, consistent, and accurate when they are reconstructed by GQI than when they are reconstructed by DTI.

## Introduction

Thirty years ago, Crick and Jones said, “If you want to clarify the principles of brain activity, you must clearly present the anatomy of your brain.” [1]. As early as 1994, Basser et al. conducted a detailed study of the structure and integrity of neurons in the brain using diffusion tensor imaging (DTI) [2]. DTI can non-invasively image the structure of the brain, which is an important step in explaining the principles of human brain activity and anatomy. Many scholars have confirmed that DTI plays an important role in the medical field [3, 4], such as preoperative localization of the pyramidal tract, the visual radiations [5], functional language areas [6], preservation of functional areas during surgery, and evaluation of prognosis. Through a large number of clinical trials, many scholars have found that DTI reconstruction of intracranial nerve fibers has several limitations. Although some axons can be revealed, sections of the brainstem still fail to achieve satisfactory visualization. Furthermore, after reconstruction, these areas show missing, incomplete, and broken fibers [7, 8]. At the same time, DTI cannot visualize fibers in tumors or regions of edema [9, 10]. To make up for these shortcomings and overcome the limitations of DTI in clinical practice, scholars have experimented with and perfected a variety of reconstruction methods. These methods can be broadly divided into model-based and model-free methods [11]. Model-based methods, such as the multiple Gaussian model, spherical harmonic decomposition model, diffusion-kurtosis model, and spherical harmonic deconvolution model, rely on a complex model to express diffuse MR signals obtained by high angular resolution diffusion imaging (HARDI) and sample data in a diffusion-encoding space called Q-space [12]. The model-free methods, also known as Q-space imaging methods, are based on the Fourier transform relationship between the diffuse MR signal and the potential diffusion displacement. They express diffuse MR signals by obtaining the orientation distribution function (ODF) of the diffusion displacement [13, 14]. Although many acquisitions and reconstruction methods have been proposed to replace DTI, such as spherical imaging (q-ball imaging, QBI) [15] and diffusion spectrum imaging (DSI) [16–18], they are insufficient to certain extents. In the process of QBI tracing, the diffused MR signal is only displaced perpendicular to the diffusion gradient vector, but the ideal diffusion displacement is in all directions [19]. Moreover, DSI requires spatial Fourier transform of the MR signal, which creates a shadow of truncated artifacts [20–22]. Juan et al. [23] have used HARDI methods such as generalized q-sampling imaging (GQI) to improve the visualization accuracy of complex nerve fibers. In this experiment, we conducted a clinical study of 5 normal participants and compare the differences between GQI and DTI reconstruction methods in displaying regions in which axons cross (crossing areas).

## Materials and methods

### Patient selection

From September 2017 to September 2018, we openly recruited 5 adult volunteers from the public (3 males, 2 females; all right-handed; age range: 30–55 years) who had no relevant clinical disease, no history of genetic diseases in related family members. Volunteers underwent nuclear magnetic scanning at the First Affiliated Hospital of China Medical University.

### Nerve fiber selection

There are thousands of nerve fibers in a person’s brain. Although some nerve fibers intersect with other nerve fibers’ ending and a lot of fibers occupy this area, artifacts and other factors will affect the imaging of nerve fibers. All nerve fibers we have chosen have a clear anatomy, which make it less controversial and convincing (Table 1). In this article, the nerve fibers we used all have intersections on the trunk of the nerve fibers, which will reduce the impact of errors and artifacts on results.

**Table 1:** Composition of crossing regions

	Crossing region 1	Crossing region 2	Crossing region 3	Crossing region 4
Selection of nerve fibers	Optic nerve	Superior cerebellar peduncles	Arcuate fasciculus, corpus callosum, pyramidal tract	Arcuate fasciculus, supplementary motor area

### MRI acquisition

MRI data were acquired with a 3.0 T whole-body MRI scanner (General Electric Medical System, GE Signa HDxt). Participants were required to remain in a fixed position in the scanner and were not allowed to move. We placed a foam pad on either side of the head to reduce head movement and used a cotton plug to reduce noise. The three-dimensional T1-weighted image obtained from the brain volume imaging (Bravo) sequence had the following imaging parameters: repetition time = 8.5 ms, echo time = 3.332 ms, inversion time = 450 ms, layer thickness = 1 mm, flip angle = 13°, excitation time = 1 ms, field of view = 240 × 240 mm^2^, voxel size = 0.5 × 0.5 × 1.0 mm^3^, and acquisition time = 215 s.

Diffusion imaging was obtained with a single-shot spin-echo planar imaging sequence with a 25-direction scan, a layer thickness of 2.4 mm, and no interlaminar gap. The coverage of the brain regions were as follows: repetition time = 8000 ms, echo time = 108 ms, flip angle = 90°, *b*-value = 1000 s/m^2^, field of view = 240 × 240 mm^2^, voxel size = 1 × 1 × 2.4 mm^3^, number of excitations = 1, 42 consecutive slices, and acquisition time = 216 s.

### Diffusion postprocessing

DSI-STUDIO software (http://dsistudio.labsolver.org/) was used to reconstruct diffusion imaging data using the DTI and GQI methods. TRACKVIS software (http://trackvis.org/) was used to track the fibers, in which the commissural tracts are shown in red and the associated fibers are shown in green. The super-to-inferior projection fibers are shown in blue.

### Image processing and analysis

We have customized a fiber-tracking diffusion-imaging tractography method that sets FA values and QA values, as well as the number of tracking fibers, before tracking. This eliminates some of the uncertainties in the tracking results, including the location and size of the seed regions of interest (ROIs). Taking these factors into account and to make our comparison more convincing, we first used the two reconstruction methods to track non-intersecting pyramidal tract fibers on one side of the brain. Knowing the performance in uncrossed areas allowed us to better explain the tracking differences in crossed areas.

We chose the pyramidal tract as the region of interest (ROI) and selected it by hand. Because the final shape, fiber number, and other aspects of the tract analysis are affected by factors such as ROI placement, DTI index, FA threshold, GQI index, QA threshold, and total number of fibers in the brain, to objectively compare DTI and GQI for crossing-fiber tracking, we use the “non-cross-fiber correction” approach to rule out the effects of other unrelated factors.

First, we loaded the Digital Imaging and Communications in Medicine DICOM data from each of the five patients into DSI Studio, renamed them, and opened them to create the “.src” file. Then we open the “.src” file to rebuild using either DTI or GQI to get the “.fib” data files. Finally, we opened the “.fib” files to track the fibers and get the DTI and GQI reconstructions. We fixed multiple parameters such as maximum angle (50°) and step size (1.2) for both DTI and GQI tracking. The pyramidal tract ROI was placed in the anterior third of the posterior limb of the internal capsule and in 3 out of the 5 mid-lateral bases of the cerebral pedunculus. The remaining factors that can affect the comparison between DTI and GQI fiber tracking are the FA and QA thresholds. We continuously adjusted the FA and QA thresholds when obtaining the DTI and GQI data so that the number of pyramidal fibers on the side of the trace was the same, the shapes were consistent, and the outcome matched the known anatomy. We found that when the FA threshold was 0.040 and the QA threshold was 0.025, the total number of fibers reconstructed by each group was 1500, and the groups had the same shape and conformed to the known anatomy. Then, under these same thresholds, we used DTI and GQI to track and reconstruct the fibers in the intracranial intersection, such as the optic chiasm, etc.

### ROI selection

#### Optic nerve

**Fig. 1:**
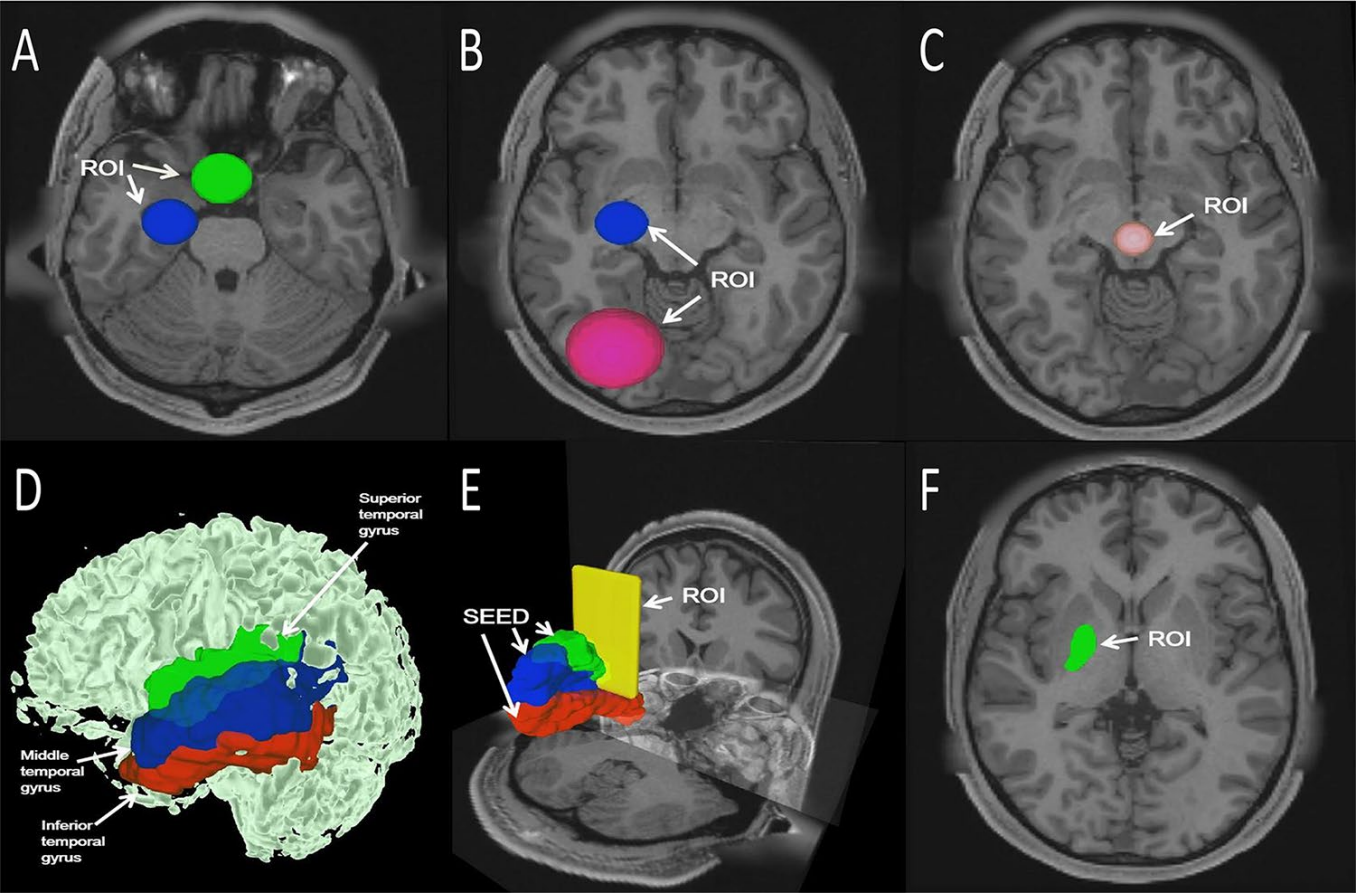
**a** ROI placed in the region of the optic chiasma is shown in green. ROI placed in the lateral geniculate body is shown in blue. **b** ROI placed in the lateral geniculate body is shown in blue. ROI placed within the occipital lobe is shown in pink. **c** ROI placed in the intersecting region of the superior cerebellar peduncles is shown in orange. **d**, **e** Green represent the Superior temporal gyrus, blue represents the Middle temporal gyrus, red represents the Inferior temporal gyrus. They were all selected as SEED. ROI placed on the coronal view at the central posterior position is shown in yellow. **f** ROI placed in the posterior limb of the internal capsule is shown in green (color figure online)

One ROI is placed at the region of the optic chiasm, and the other is placed in the lateral geniculate body (Fig. 1a). ROIs for the optic radiation: one is placed in the lateral geniculate body and another is placed within the occipital lobe (Fig. 1b).

#### Superior cerebellar peduncles

The ROI was placed at the level between the midbrain peduncles and the superior cerebellar peduncles in the axial phase. Specifically, the ROI included the junction of the two superior cerebellar peduncles at the level of the lower part of the midbrain, at the level of the cerebral peduncle, in the midbrain, and in front of the mesencephalic aqueduct (Fig. 1c).

#### Arcuate fasciculus

We placed the seeding area on the superior temporal gyrus, middle temporal gyrus, and Inferior temporal gyrus, and then placed a mask ROI on the coronal view at the central posterior position (Fig. 1d, e).

#### Pyramidal tract

We placed the ROI in the posterior limb of the internal capsule and tracked the fibers (Fig. 1f).

#### Corpus callosum

Using the ‘Select’ command on the median sagittal plane, we selected the entire corpus callosum as the ROI.

#### Supplementary motor area

ROI was placed in the Broca area, we selected nerve fibers on the path of the supplementary motor area manually.

## Results

DSI-STUDIO software was used to perform GQI and DTI reconstruction on each patient’s raw data then we compared the results of the reconstructions. In general, the fiber structure based on DTI imaging of the crossing areas was missing, broken, incomplete, and had artifacts. However, the fibers visualized by GQI were relatively complete, accurate, and conformed to the anatomical structure. Additionally, the GQI imaging was relatively smooth. Here, we describe our most significant findings when applying DTI and GQI to reconstruct the nerve fibers (Table 2).

**Table 2:** Comparison of data between two reconstruction methods

	Number of tracking fibers				Whether there is an obvious intersection			
	Crossing region 1	Crossing region 2	Crossing region 3	Crossing region 4	Crossing region 1	Crossing region 2	Crossing region 3	Crossing region 4
GQI	16,220	1907	5503	6691	Y	Y	Y	Y
DTI	15,907	2256	5324	6559	N	N	N	N

QA threshold: 0.025, FA threshold: 0.040

**Fig. 2:**
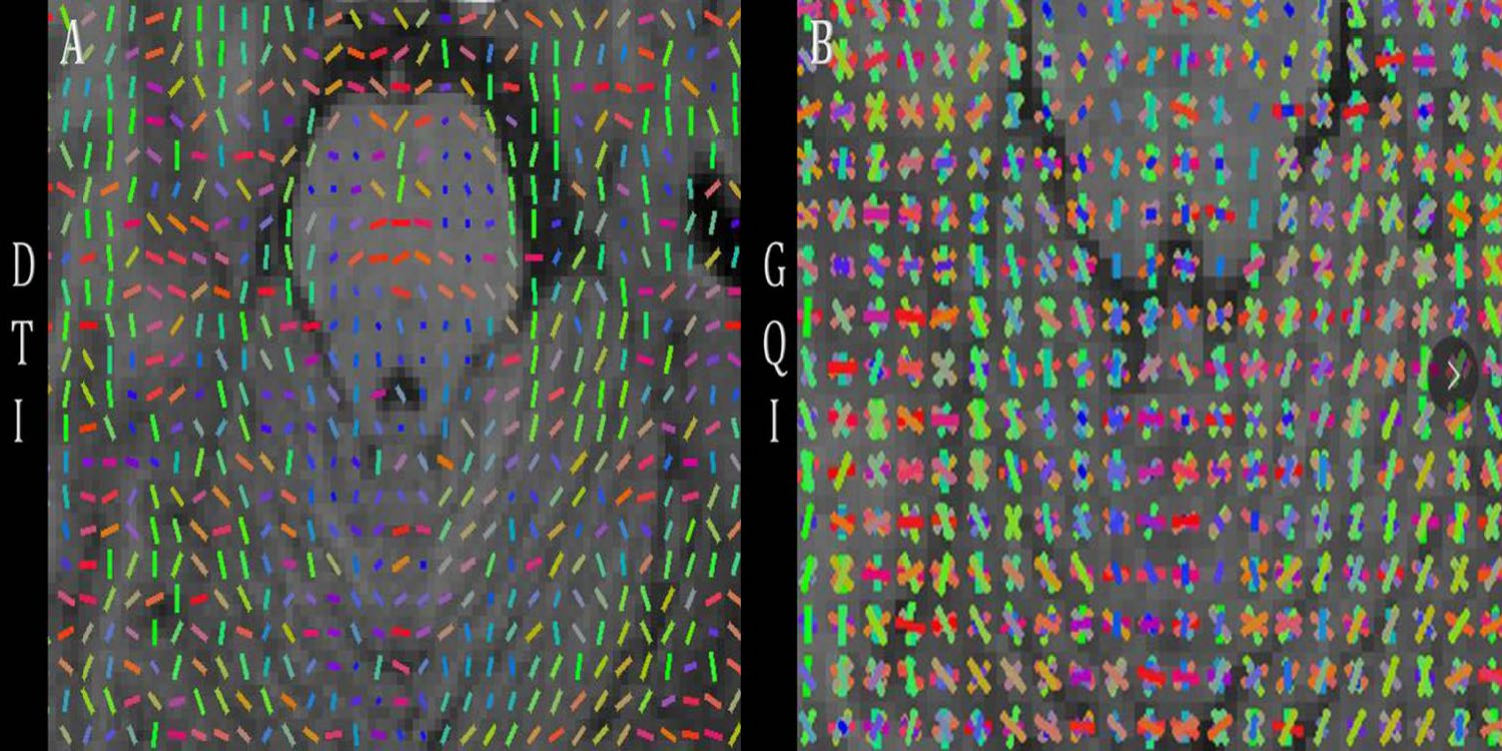
**a**, **b** The FA maps selected at the level of the superior cerebellar peduncle after DTI and GQI reconstruction of the raw data. **a** The FA maps reconstructed by DTI. **b** The FA maps reconstructed by GQI. Fibers in **a** and **b**, respectively, are color coded by direction (eigenvector orientation): red, left to right; green, anterior to posterior; and blue, inferior to superior. Intermediate colors reflect intermediate (“oblique”) directions (color figure online)

We performed the FA maps at the level of the superior cerebellar peduncles. It can be seen that although only a single bundle of fibers can be traced in a voxel after DTI reconstruction (Fig. 2a), two or more bundles of fibers can be imaged in one voxel after GQI reconstruction (Fig. 2b). This confirms that GQI is superior to DTI in displaying intracranial intersections.

### Nerve fiber crossing region 1

**Fig. 3:**
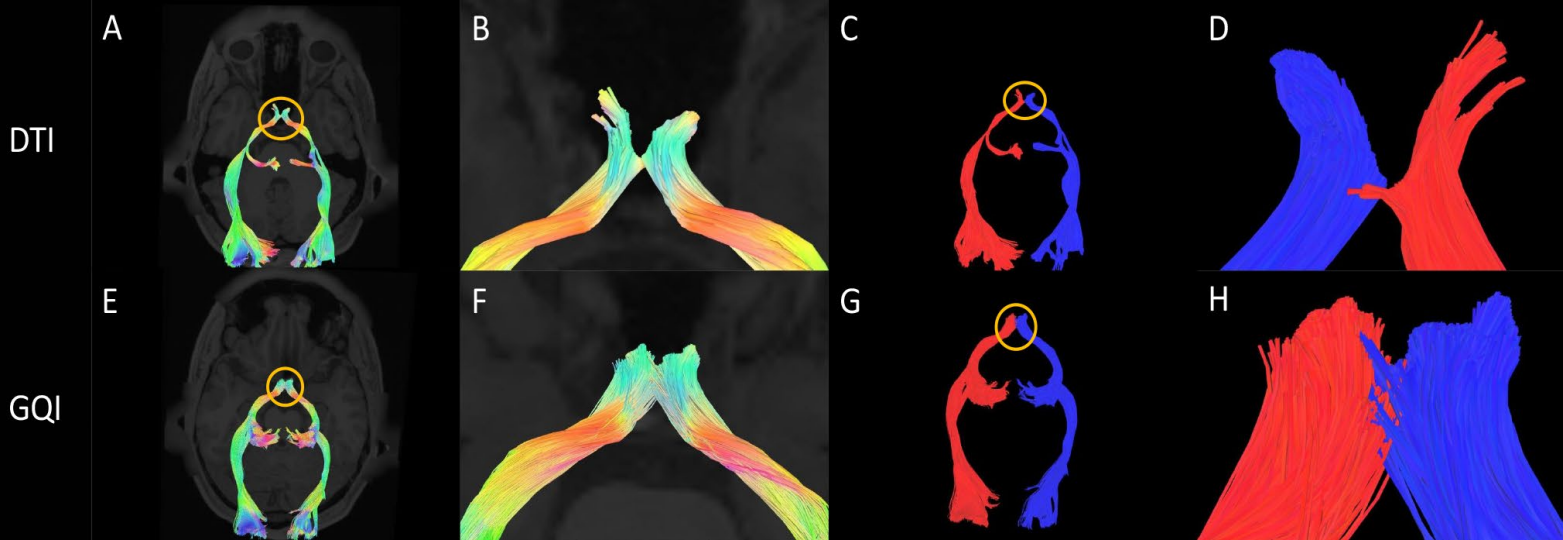
**a** Image of the optic nerve reconstructed by DTI. The circle is the optic chiasm. **b** A close-up view of the circled section in **a**. **c** The left and right sides of the optic nerve are marked with different colors according to the anatomy. The right optic nerve is shown in blue and the left optic nerve is shown in red. The circle is the optic chiasm. **d** A close-up view of the circled section in **c**. **e** Image of the optic nerve reconstructed by GQI. The circle is the optic chiasm. **f** A close-up view of the circled section in **e**. **g** The left and right sides of the optic nerve are marked with different colors according to the anatomy. The right optic nerve is shown in blue and the left optic nerve is shown in red. The circle is the optic chiasm. **h** A close-up view of the circled section in **g** (color figure online)

Reconstructions of the optic chiasm fibers can be seen in Fig. 3. The left and right optic nerve fibers are represented by red and blue, respectively. In this way, the fiber structure in the intersecting region can be observed more clearly (Fig. 3). It can be seen from the figure that after reconstruction using DTI (Fig. 3f), the nerve fibers in the intersecting region appear incomplete, missing, and broken, and there is a decrease in the number of nerve fibers around the chiasm and an incomplete number of tracing fibers. After tracking, we marked the different colors of the nerve fibers on both sides and found that the intersection was a pseudo-crossing and did not conform to the anatomy. In contrast, after the reconstruction by GQI, it can be seen that the fibers in the optic chiasm area show the normal crossing shape (Fig. 3h), the left side is red, and the right side is blue, which is consistent with anatomical knowledge.

### Nerve fiber crossing region 2

**Fig. 4:**
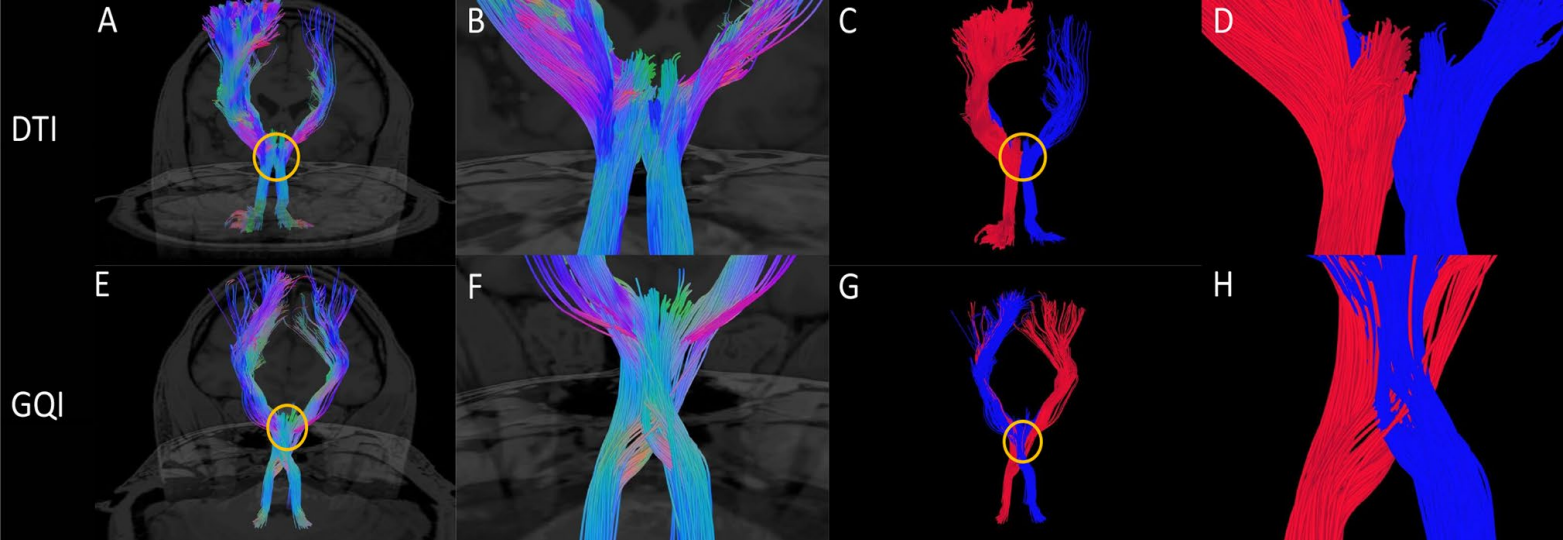
**a** Image of the superior cerebellar peduncle reconstructed by DTI. The circle is the crossing region of the superior cerebellar peduncle. **b** A close-up view of the circle section in **a**. **c** The left and right sides of the superior cerebellar peduncle are marked with different colors according to the anatomy. The right superior cerebellar peduncle is shown in blue and the left superior cerebellar peduncle is shown in red. The circle is the optic chiasm. **d** A close-up view of the circle section in **c**. **e** Image of the superior cerebellar peduncle reconstructed by GQI. The circle is the crossing region of the superior cerebellar peduncle. **f** A close-up view of the circled section in **e**. **g** The left and right sides of the superior cerebellar peduncle are marked with different colors according to the anatomy. The right superior cerebellar peduncle is shown in blue and the left superior cerebellar peduncle is shown in red. The circle is the optic chiasm. **h** A close-up view of the circled section in **g** (color figure online)

After reconstruction, according to the anatomy of the superior cerebellar peduncle, we labeled the left and right axons in different colors to observe the fibers in the crossing area. The nerve fibers running on the right side are shown in blue and those on the left side are shown in red (Fig. 4). We present the results of the two reconstruction methods on an image for easy comparison analysis. As it can be seen (Fig. 4c, d), the fibers that should have been crossed appear in an approximately parallel shape after DTI reconstruction, and obviously do not conform to the anatomy due to artifacts. Moreover, the number of fibers in the crossing area is small after DTI reconstruction, and there are cases of incomplete tracking and fracture. In contrast, the number of fibers in the crossing area after GQI reconstruction is higher (Fig. 4g, h) and the fibers travel continuously with few occurrences of fracture, loss, or incompleteness. Moreover, after GQI reconstruction, it can be seen that the fibers in the lower part of the mesencephalon cross to the opposite side and are distributed in the mesencephalon and diencephalon.

### Nerve fiber crossing region 3

**Fig. 5:**
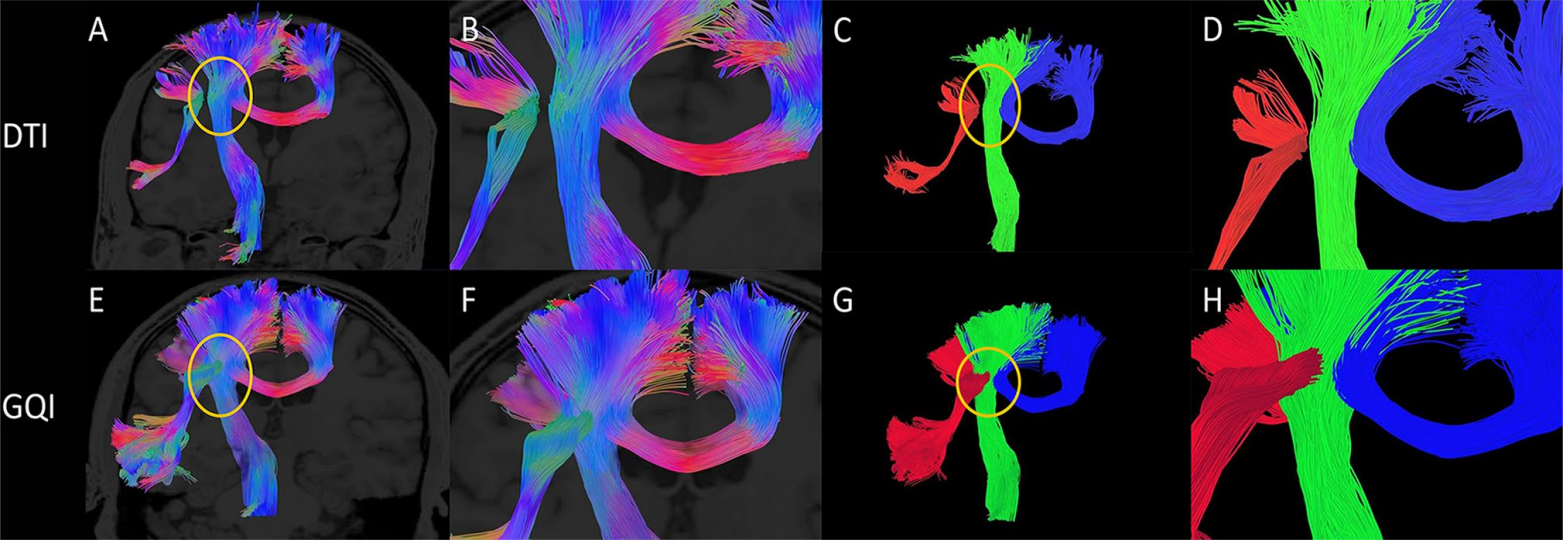
**a** Image of pyramidal tract, the corpus callosum, and the arcuate fasciculus reconstructed by DTI. The circle is the crossing region for the pyramidal tract, the corpus callosum, and the arcuate fasciculus. **b** A close-up view of the circled section in **a**. **c** Different tracts are marked with different colors: the pyramidal tract is shown in green, the corpus callosum is shown in blue, and the arcuate fasciculus is shown in red. The position of the circle is the area where the three tracts of fibers intersect. **d** A close-up view of the circled section in **c**. **e** Image of pyramidal tract, the corpus callosum and the arcuate fasciculus reconstructed by GQI. The circle is the crossing region for the pyramidal tract, the corpus callosum and the arcuate fasciculus. **f** A close-up view of the circled section in **e**. **g** Different fiber tracts are marked with different colors: the pyramidal tract is shown in green, the corpus callosum is shown in blue, and the arcuate fasciculus is shown in red. The position of the circle is the area where the three tracts intersect. **h** A close-up view of the circle section in **g** (color figure online)

We performed GQI and DTI reconstruction on the pyramidal tract, arcuate bundle, and corpus callosum of the five participants. The corpus callosum is shown in blue, the pyramidal tract is shown in green, and the arcuate fasciculus is shown in red (Fig. 5). We focused on the imaging of three fiber bundles in the crossing area. It can be seen that in the DTI reconstruction, the three bundles have only a few fibers crossing in the crossing area (Fig. 5c, d). Meanwhile, the arcuate fasciculus is clearly separated from the pyramidal tract and the corpus callosum. Moreover, there is no case in which three fibers cross at the same time. Meanwhile, the fibers in the crossing area are also broken, incomplete, and missing. After looking at the visualization rebuilt through DTI, one would think that the three bundles do not cross. However, the story is quite different after GQI reconstruction. It can be clearly seen that the paths of these three fiber bundles obviously intersect and that there are not only two fiber bundles in the intersection but all three. The fiber cross together, and the distribution of crossed fibers is relatively tight, and the number is large. Compared with DTI, GQI reconstruction shows that the fibers in the intersecting area are numerous, intact, and smooth (Fig. 5g, h). Thus, this method compensates for the shortcomings of DTI imaging in fiber-crossing areas.

### Nerve fiber crossing region 4

**Fig. 6:**
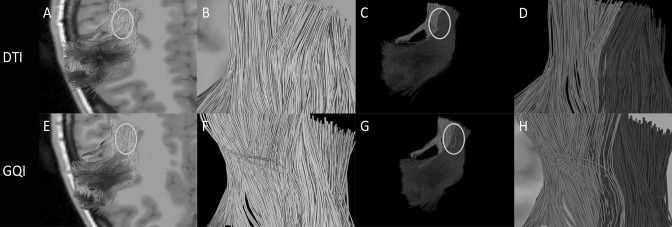
**a** Image of arcuate fasciculus and the supplementary motor area (SMA) reconstructed by DTI. The circle is the crossing region for the SMA and the anterior part of arcuate fasciculus. **b** A close-up view of the circled section in **a**. **c** Different tracts are marked with different colors: the anterior part of arcuate fasciculus is shown in red, the SMA is shown in blue. The position of the circle is the area where the two tracts of fibers intersect. **d** A close-up view of the circled section in **C**. **e** Image of arcuate fasciculus and the SMA reconstructed by GQI. The circle is the crossing region for the SMA and the anterior part of arcuate fasciculus. **f** A close-up view of the circled section in **e**. **g** Different tracts are marked with different colors: the anterior part of arcuate fasciculus is shown in red, the SMA is shown in blue. The position of the circle is the area where the two tracts of fibers intersect. **h** A close-up view of the circle section in **g** (color figure online)

After reconstruction, we can get two different results in two reconstruction methods. Red shows the movement of the anterior part of arcuate fasciculus, blue shows the movement of the supplementary motor area (SMA) (Fig. 6). We present the results of the two reconstruction methods on an image for easy comparison analysis. After reconstruction by DTI, these two kinds of nerve fibers go in a parallel way and there is no obvious intersection in the path of the two nerve fibers (Fig. 6c, d). However, we can observe that there are a lot of intersecting areas between the two nerve fibers after reconstructed by GQI (Fig. 6g, h).

## Discussion

There are thousands of nerve fibers in the human brain, which can be divided into two groups based on whether they cross other fibers or not. This paper focuses on the crossing fibers present in the brain. There are five major fiber intersections in the brain, including the optic nerve, the superior cerebellar peduncle, the pyramidal tract, the corpus callosum, and the arcuate fasciculus. In clinical cases, patient data is often reconstructed using DTI. However, our results clearly show that DTI imaging at fiber intersections is far from perfect. Because DTI was developed from diffusion-weighted imaging (DWI) [24], it uses anisotropic imaging of water molecule diffusion motion along with processing technology to track white matter fibers and reflect the direction of their anatomical connectivity, as well as to detect tissue microstructure, all to study human brain function. DTI is similar to DWI, but has a longer acquisition time and provides more accurate quantitative information [24]. However, DTI can only distinguish the direction of individual fibers within each voxel. Meanwhile, the traced images can have artifacts that arise from averaging the volume of complex neural structures. Additionally, partially averaging the volume of complex nerve structures fails to solve complex structures such as fiber intersections and branching fiber patterns [11], and the origin and destination of fibers cannot be accurately determined [20, 25, 26]. Therefore, it is impossible to visualize the intersection of nerve fibers and the complex multi-branch structure of axons in the brain. At the same time, because DTI algorithms are primarily energy-minimum methods combined with linear extension data algorithm, the algorithms also have various limitations and obvious artifacts in the processing of crossing fibers and complex intracranial fibers. To a certain extent, it limits the ability of DTI and its corresponding parameters to be an effective guide in clinical setting. The GQI technique we examined here is a new way to reconstruct fiber tracts in the brain. Yeh et al. [19] derived a new relationship between spin distribution function (SDF) and MR based on the Fourier transform the relationship between the diffuse MR signal and the spin diffusion displacement. The SDF can be obtained in a shell sampling scheme in QBI or in a grid sampling scheme such as is found in DSI. The SDF value is calculated by scaling the average propagation medium and unifying the SDF size between different voxels using a density function. Because SDF defines the proportion of different voxels, the SDF obtained by GQI contains a certain meaning for all voxels [20]. Due to these factors, voxels are comparable [19]. Because the traditional ODF is the probability distribution of displacement, the newly developed SDF represents the distribution of spins in the diffusion process [19]. This principle makes SDF more advantageous in tracking nerve fibers. GQI is the result of mathematical simplification that combines Fourier transform with ODF calculation, thus deducing the direct relationship between the spread signal and SDF. This method avoids the Fourier transform and subsequent interpolation on the grid data points [27]. Meanwhile, Yeh et al. defined an indicator called quantitative anisotropy (QA) to recalculate the population of spins in a particular direction. Compared with the FA value, the biggest advantage of QA is that it not only distinguishes one fiber in the voxel, but the fiber group in the voxel can be marked so that the reconstructed image is more accurate [19, 28]. Therefore, in addition to data acquisition schemes like QBI and DSI, such as single-shell, multi-shell, or grid sampling schemes, GQI can also provide crossing-fiber orientation and quantitative information to perfectly solve complex crossing-fiber imaging problems [19, 29]. In the process of tracking reconstruction, the ideal diffuse MR signal is not simply the displacement of the diffusion gradient vector as QBI, but the diffusion displacement in all directions [19]. To form a diffusion density function (PDF), DSI requires not only spatial Fourier transform of the MR signal, but also numerical estimation. Because truncation artifacts are often encountered in Fourier variations [20–22], a Hannning filter is often required to smooth the probability density function, which results in a bias in the reconstructed information to some extent [30, 31]. The GQI introduced in this paper not only has the advantages of these reconstruction methods but to some extent also solves the problems caused by these methods.

We present our results applying GQI to investigate the structure of the normal human brain. GQI represents a significant improvement in MR-based fiber tracking techniques. The resolution of the crossing problems is illustrated here with the accurate replication of known neuroanatomical features such as the optic chiasm, the superior cerebellar peduncles, the pyramidal tract, the corpus callosum, and the arcuate fasciculus.

Wang et al. used GQI and DTI reconstruction in 5 patients with brain tumors [14]. The patients were scanned, and tracts were reconstructed by the two methods before operation. They found that DTI failed to meet the ideal standard for fibers within edema, and in fact could not image any fibers within them. GQI clearly showed the location and number of fibers within areas of edema. After the operation, the edema subsided and GQI and DTI reconstruction were performed again. At this time, the nerve fibers in the original preoperative edema area were visualized after DTI reconstruction. Studies have shown that GQI can also better display nerve fibers in areas of cerebral edema [14].

In this study, we confirmed that GQI can visualize nerve fibers in crossing areas more completely and continuously than DTI by comparing reconstructions of four intersections in five healthy volunteers. Moreover, GQI can better determine the location of intracranial nerve-fiber intersections. It is particularly important to determine the anatomical structure of some nerve fibers by defining the structure of nerve fibers in the cross region. When the anatomical structure of the nerve fibers is clear, we can judge the severity of the disease and the choice of surgical methods by preoperatively identifying the relationship between the tumor and brain structure. Looking at the optic chiasm and the crossing region of the superior cerebellar peduncles, it can be clearly seen that when DTI is used, there is a pseudo-crossing phenomenon in the crossing region, and there are artifacts of fiber fracture. In Crossing Region 3, imaging the pyramidal tract, the arcuate fasciculus, and the corpus callosum by GQI allowed all three intersections to be clearly located. In contrast, DTI could not determine whether there were any common intersections between the three fiber bundles in their respective paths. This study used these four aspects to confirm the superiority of GQI imaging in fiber-intersection regions. Although DTI is widely used in clinic, the advantages of GQI are clearly shown in this article. Therefore, we believe that GQI can better display the fibers in intracranial intersections and is a promising technique for disease assessment, especially when it occurs in the vicinity of fiber- crossing regions.

## Limitations

Although GQI is better than DTI at quantifying and displaying fibers in the crossing areas, there are still some obvious limitations in this reconstruction method. These limitations are found in almost all MRI methods. First, the process of track reconstruction is determined by researchers and the reconstruction result is closely related to FA value, QA value, angle threshold, slice thickness, and sample number [32]. because differences in parameter selection during the reconstruction process leads to differences in the nerve fibers being tracked, the practicality of the diffusion imaging methods we use is somewhat controversial. Second, the number and direction of the fibers we track depend on the size and location of the ROI. Different ROIs lead us to track different fibers, which makes the process of selecting ROIs more complicated and full of uncertainty [15]. Third, if we meet the balanced needs of the sampling scheme, then the measured SDF used by GQI does not necessarily guarantee the correct result [19].

This study was also limited by the type of nerve fibers being tracked and the location and extent of the intersections. First, the numbers of some types of fibers were relatively small, which led to an even fewer number of fibers in the crossing areas. Second, the crossing areas for many nerve fibers are obviously larger than those of others. Therefore, in the process of ROI selection, whether the ROI can be accurately set determines the accuracy of the results. Finally, fibers different among people to some extent, which leads to deviations in the tracking process. For further research, more samples are required to confirm the ability of GQI to image nerve fibers in all crossing regions of the brain.

## Conclusion

The GQI-reconstruction method is still new, and there are many limitations in its ability to visualize nerve fibers in crossing areas. However, compared with other reconstruction methods such as DTI, GQI performs more accurately, and its advantages are quite obvious. As the fiber paths were clear, this is a positive sign that further studies should be made that increase the sample size and definitively show the advantages of GQI. If this can be achieved, the scope of GQI application will increase and GQI will likely become an auxiliary tool for neurosurgery in the future.
